# Study of the Mandibular Bone Microstructure and Blood Minerals Bioavailability in Rainbow Trout (*Oncorhynchus mykiss,* Walbaum 1792) from Freshwater

**DOI:** 10.3390/ani12121476

**Published:** 2022-06-07

**Authors:** Karina Godoy, Cristian Sandoval, Carlos Manterola-Barroso, Claudio Vásquez, Noelia Sepúlveda, Mariana Rojas, Luis A. Salazar

**Affiliations:** 1 Programa de Doctorado en Ciencias Morfológicas, Facultad de Medicina, Universidad de La Frontera, Temuco 4811230, Chile; karina.godoy@ufrontera.cl (K.G.); mrojasr@u.uchile.cl (M.R.); 2 Centro de Biología Molecular y Farmacogenética, Departamento de Ciencias Básicas, Facultad de Medicina, Universidad de La Frontera, Temuco 4811230, Chile; claudio.vasquez@ufrontera.cl; 3 Núcleo Científico y Tecnológico en Biorecursos (BIOREN), Universidad de La Frontera, Temuco 4811230, Chile; carlosignacio.manterola@ufrontera.cl (C.M.-B.); noelia.sepulveda@ufrontera.cl (N.S.); 4 Escuela de Tecnología Médica, Facultad de Salud, Universidad Santo Tomás, Osorno 5310431, Chile; cristian.sandoval@ufrontera.cl; 5 Departamento de Ciencias Preclínicas, Facultad de Medicina, Universidad de La Frontera, Temuco 4811230, Chile; 6 Programa de Anatomía y Biología del Desarrollo, ICBM, Facultad de Medicina, Universidad de Chile, Santiago 8330015, Chile

**Keywords:** micronutrient, bone fish elemental composition, electron microscopy, X-ray spectroscopy (EDX), thermogravimetric analysis

## Abstract

**Simple Summary:**

Bone deformities in the axial skeleton represent a frequent bone pathology in farmed salmonids, affecting the quality of life and even increasing mortality. Bone deformation can affect the formation, repair, and regeneration of inorganic-bone components and is associated with diet, culture conditions, and genetics. If diet and culture conditions are factors in the development of bone deformity, wild fish lack them and present fewer deformations than farmed fish. Hence, we studied mandibular bone microstructure using variable pressure scanning electron microscope (VP-SEM) coupled to EDS detector. Two groups of smolt rainbow trout were analyzed: Group 1, farmed fish with a control diet, and Group 2, wild fish without a control diet. We observed that serum protein levels remained within normal ranges. However, the calcium and phosphorus ratio was not the same in blood as in bone; phosphorus deficiency was more critical because it forms other structural molecules such as nucleic acid, phosphoproteins, phospholipids, and high-energy phosphates. Consequently, the microstructure in wild fish showed a more significant number of pores and microfractures per area, which was detrimental to the biomechanical properties of the bone.

**Abstract:**

Farmed salmonids show alterations in bone structure that result in skeletal deformities during formation, repair, and regeneration processes, with loss of mineralization at the level of the axial skeleton, mainly the head and spine, affecting their quality of life and even causing death. Despite improving factors, such as farming conditions, diets, and genetics, bone alterations appear more frequently in farmed fish than in wild fish. Thus, we used SEM-EDX, and TGA-DSC to study bone mineralization in farmed and wild rainbow trouts. As expected, we found significant differences in the nutritional parameters of farmed and wild fish (*p* < 0.05). Microstructural analyses indicated that farmed fish have a more robust mineral structure (*p* < 0.05), confirming the differences in mineralization and microstructure between both groups. However, the mechanisms regulating absorption and distribution in the organism and their effect on bone mineralization remain to be known. In our study, the combined use of techniques such as SEM-EDX and TGA-DSC allows a clearer assessment and detailed characterization beneficial to understanding the relationship between diet control and bone microstructure.

## 1. Introduction

Rainbow trout (*Oncorhynchus mykiss)* is a teleost fish with cellular bone [1], belonging to the salmonid family (Salmonidae). Rainbow trout can be found in free form in rivers or lagoons (wild), or raised in aquaculture, mainly in North and South America, Asia, and Europe. Despite the development of salmon farming and the conditions given to farmed fish, fish culture reduces natural selection, with the consequent onset of bone alterations affecting development [2,3,4]. The more frequent deformations locate in the axial skeleton (head and spine) and consist of jaw deformities associated with specimens fed with low-vitamin diets (A and C), proteins, and minerals, primarily phosphorus (P) [5,6,7,8,9].

Bone alterations in fish have been described in association with genetic, environmental, and nutritional factors, thus establishing that the origin of bone alterations in salmonids is multifactorial [7,9,10,11,12,13]. Therefore, the correct identification of the determinant component is crucial for a better understanding of the process, preventing damage to the species, improving the efficiency of the productive chain, and avoiding economic losses [2,4,8,14]. Genetic factors affecting salmonid bone development are mainly related to gene expression alterations in signaling pathways for osteogenesis, affecting one or several genes [15,16]. Environmental factors refer to growing conditions, such as photoperiod, temperature, light intensity, dissolved oxygen, pH, salinity, carbon dioxide, water flow, presence of heavy metals, fungicides, pesticides, and crop intensification [3,13,17,18,19]. Another factor to consider is the stress associated with culture conditions, which can lead to loss of bone mineralization by induction of osteoclast activity [20]. Nutritional factors are directly related to protein consumption, amino acids (tryptophan and lysine), fatty acids, phospholipids, minerals, and vitamins (A, C, D, E, and K) considered fundamental for the maintenance, growth, and renewal of tissues. In this sense, an adequate intake of minerals such as Magnesium (Mg), Manganese (Mn), Zinc (Zn), Fluorine (F), Iron (Fe), Calcium (Ca), and Phosphorus (P) can reduce the risk of deformations. Of all of the above, P is the most relevant due to its contribution to forming the hydroxyapatite (HPA) crystal, the main component of the bone inorganic matrix [7,21,22,23,24,25,26]. In addition, an excess in Fe or Zn can be detrimental to bone homeostasis and overall fish health. [27,28].

Homeostasis between Ca and P at the systemic level is critical for HPA production [Ca_10_(PO4)_6_(OH)_2_], a mineral component playing a key role in bone strength [1,9,29,30]. The Ca/P ratio has been proposed as a reference measure to assess inorganic bone homeostasis. Thus, Ca/P ratios with values of 1, 1.65–1.90, and 2 have been reported, the latter being the most widely used because it represents the stoichiometry of these elements in the HPA crystal [7,8,9,26,31,32]. In fish, it has been reported that deficient dietary P levels result in low P uptake, increased Ca/P ratio, decreased growth, deformed opercula, and altered bone ultrastructure due to decreased mineralization of hard tissues such as bone and cartilage [8,9,26,31]. Recent studies point out a dependence of the mineralization process and bone development on Mg content due to its action as a second messenger in bone tissue regeneration and Ca absorption through its role in Vitamin D activation and calcitonin hormone stimulation [33,34]. Fe is widely known for its role in oxygen (O) transport by being a structural part of hemoglobin (Hb) and myoglobin [35]. In addition, it acts as an enzymatic cofactor in alpha-1-hydroxylase, which is involved in vitamin D metabolism and activation and in alpha-ketoglutarate dependent hydroxylases playing a role in the synthesis and control of collagen expression [35,36]; or in prolyl hydroxylase (PDH) that has effects on osteoblast and osteoclast activity [35,37]. Several studies report that low Fe availability produces the following: (1) affects the bone structure, as well as the expression of biomarkers of bone metabolism [35,38]; (2) accompanies dietary Ca restriction, affecting the bone structure [35,39]; and (3) decreases the expression of N-terminal intact procollagen 1 (P1NP) and increases the levels of parathyroid hormone and acid phosphatase produced by osteoclasts, increasing bone resorption, which decreases Ca and P levels, ultimately generating low or imperfect mineralization [40].

Blood screening for clinical purposes in salmonids is frequently used to assess general physiological status, metabolism, and evaluation of changes or new diets and conditions, representing an important tool for evaluation, diagnosis, and prognosis of fish health status as previously described [41,42,43,44,45]. Furthermore, complementing these studies with microstructural analyses of bone (physicochemical techniques) is fundamental to understanding bone structuring. Therefore, we studied bone mineralization and microstructure using variable pressure scanning electron microscope (VP-SEM) coupled to EDS detector in smolt rainbow trouts (*Oncorhynchus mykiss*) with and without nutritional control (farmed vs. wild fish).

## 2. Materials and Methods

### 2.1. Specimens Sampling

Juvenile rainbow trout (*Oncorhynchus mykiss*), approximately 50 cm in size (maximum), were selected from fish farming and wildlife (considered the relevant difference was in the color of the skin and size). The capture of wild fish was carried out by personnel authorized for this by Servicio Nacional de Pesca y Acuicultura (SERNAPESCA, N °20.256 of 2008, Art. 6); while the capture of farmed fish was realized by authorized fish farming personnel. In both cases, fly fishing to catch juvenile fish was used [46]. In total, 10 fish were obtained for analysis and distributed into two groups. Group 1: 6 specimens from non-intensive fish farming, clinically healthy, for which regular swimming, vision, no fin erosions, no externally visible lesions or tumors on the fish body, and no presence of bacterial or parasitic diseases; and Group 2: 4 wildlife specimens obtained from the pre-cordillera lake, no fin erosions, no externally visible lesions or tumors on the fish body, no presence of bacterial or parasitic diseases. We used Benzocaine 20% (Veterquímica S.A., Santiago, Chile) 30–40 ppm to obtain blood samples. Subsequently, they were euthanized by overexposure to the anesthetic using a time of more than 10 min. Blood samples and specimens were stored at 0 °C in a container with ice until they reached the laboratory. The Scientific Ethical Committee of Universidad de La Frontera approved the experimental protocol (N °061_20).

### 2.2. Sample Processing

We centrifuged blood samples at 1500 rpm; serum was separated from the cellular component and stored at −20 °C until further analysis. The euthanized specimens were decapitated and washed with physiological saline (NaCl 0.9% *w*/*v*). The jaws were separated from the head and treated with deionized water at 60–70 °C for about 20 min to facilitate the removal of skin, muscle, connective tissue, and other organic components, leaving free the inorganic component without altering its structure. The jaws were fixed in buffered formalin (10% in 1X PBS; pH 7.4) until analysis.

### 2.3. Quantification of Serum Metabolites

Serum samples were thawed at room temperature. The following parameters were quantified: Total Proteins, Albumin, Globulins, Glucose, Enzymatic Activity (Phosphatases, and Creatine Kinase), and microelements (Calcium, Phosphorus, Iron, and Magnesium), according to the protocol of the manufacturer (HUMAN Diagnostics, Wiesbaden, Germany) in a multimodal Synergy HT reader (BIOTEK, Winooski, VT, USA).

### 2.4. Scanning Electron Microscopy (VP-SEM) Microstructure and Semi-Quantitative Elemental Microanalysis in Mandibular Bone

#### 2.4.1. Microstructural Analysis

The fixed mandibular bone samples were washed with distilled and deionized water three times and dried in an oven at 25 °C for 24 h. The dried sample was adhered to the sample holder with double-sided carbon tape. The separated sample was visualized in the middle area of the mandibular bone, including the teeth. Visualization was performed using a chemical contrast detector (Backscatter, BSE) in variable pressure without any further sputtering under the following parameters: 10 KV energy, 20 Pa pressure, WD 10 mm in Scanning Electron Microscope (Hitachi SU3500, Tokyo, Japan), and images were acquired and analyzed with Hitachi software controller and ImageJ 1.53k Java 1.8.0_172 Software (Wayne Rasband and contributors, National Institutes of Health, Betheda, MA, USA).

Bone porosity was determined using the ImageJ tool as follows: (1) chose an area of the control picture (farmed fish) with no borders or faults; (2) trimmed the section and adjusted the threshold to visualized regions, bright (bone), and dark (pores); (3) recorded the cut-off value on the dark areas and reproduced it to all photos to be analyzed; (4) chose the analyze particle tool (size and circularity); and, lastly, (5) began running and registering values in all photographs. ImageJ 1.53k Java 1.8.0 172 Software (Wayne Rasband and contributors, National Institutes of Health, Bethesda, MA, USA).

#### 2.4.2. Semi-Quantitative Elemental Microanalysis

The quantification and elemental distribution were coupled to the X-ray energy dispersive spectroscopy detector (EDS). We chose three quantification points for each region of interest (ROI). The acquisition was performed under the following parameters: applied energy 15KV, pressure 20 Pa, WD 10 mm using Scanning Electron Microscope (Hitachi SU3500, Tokyo, Japan) coupled to XFlash^®^ Detector 410 and Quantax Esprit 1.8.1 Software controller (Bruker, Germany).

### 2.5. Thermogravimetric Analysis in Mandibular Bone (TGA-DSC)

Whole mandibular bone samples, including teeth, were dried in an oven for 48 h at 40 °C. Subsequently, they were reduced to a fine powder by pulverization with a mortar and pestle. Approximately 20 mg of samples were subjected to two thermogravimetric analyses: first, from 25 °C to 850 °C (10 °C per minute rate) to evaluate bone sample stability in the air atmosphere by monitoring weight loss, and second, differential thermal behavior (DSC) from 25 °C to 850 °C (50 °C per minute rate) (Thermogravimetric Analysis TGA/DSC STA 6000, Perkin Elmer, Waltham, MA, USA).

### 2.6. Statistical Analysis

The normality of the data was analyzed using the D’Agostino–Pearson test for descriptive statistics. Differences between groups were analyzed with the Mann–Whitney U test. The value *p* < 0.05 was considered statistically significant (GraphPad Software, version 9.0, San Diego, CA, USA).

## 3. Results

### 3.1. Bioavailability of Metabolites and Microelements

We analyzed blood samples for metabolites informative of the nutritional status (proteins, enzymes, and glucose) and microelements directly involved in promoting the bone mineralization. Regarding total protein and albumin content, we observed lower values for wild fish (47.0 g/L; *p* < 0.001 and 18.1 g/L; *p* < 0.001, respectively) than farmed fish (Table 1). Globulins were obtained by the difference between total protein and albumin content, with the lowest content observed in wild fish (28.9 g/L; *p* = 0.053). Regarding enzymatic activities, all measurements in wild fish showed significant differences with farmed fish: creatine kinase (2192 U/L; *p* = 0.024), alkaline phosphatase (594 U/L; *p* < 0.001), acid phosphatase (13.3 U/L; *p* < 0.001), serum glucose (3.43 mmol/L; *p* < 0.001).

Quantification of serum microelements showed no significant differences in Ca levels. However, we observed the main differences in P and Mg levels (Table 2).

### 3.2. Microstructural and Microelemental Analysis in Mandibular Bone

**Figure 1:**
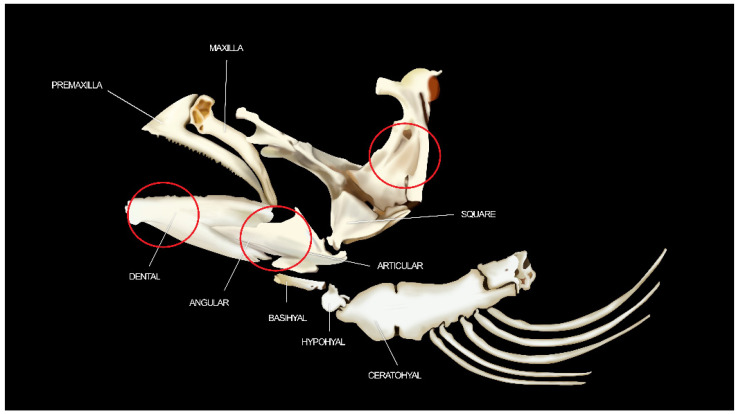
Diagram of the region of interest of the salmonid mandible. The mandible is formed by the dentary bone, the angular bone that enters the dentary, articular and retroarticular. In addition, the square bone is observed completing the premaxillary and maxillary articulation modified [47].

We focused the microstructural analysis of the fish on the jaw, between dentary to opercular (red region), and including the teeth (Figure 1).

**Figure 2:**
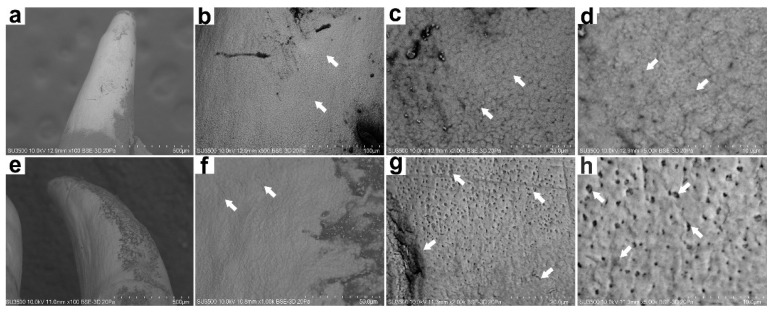
Microstructure of rainbow trout (*Oncorhynchus mykiss*) teeth. Farm fish at 500×, 1000×, 2000× and 5000× magnification (**a**–**d**). Wild fish at 500×, 1000×, 2000×, and 5000× magnification (**e**–**h**). Backscatter detector (BSD) image. Scanning Electron Microscope SU3500, Hitachi, Tokyo, Japan.

In terms of tooth morphology, there are no apparent differences between farmed and wild fish (Figure 2). Indeed, in both cases, we could observe that the teeth are conical, sharp, and curved (Figure 2a,e). However, when analyzing the surface microstructure of farmed fish teeth, it was observed that the teeth present minor flaws on their surface. The microstructure tends to be more homogeneous in the inorganic matrix with the formation of small structures characteristic of HPA crystal formation (Figure 2b,d). In contrast, a more significant amount of surface flaws in wild fish teeth is evident. We even observed large porosity, which may correspond to alterations or loss of the surface layer of the tooth (Figure 2f,h).

**Figure 3:**
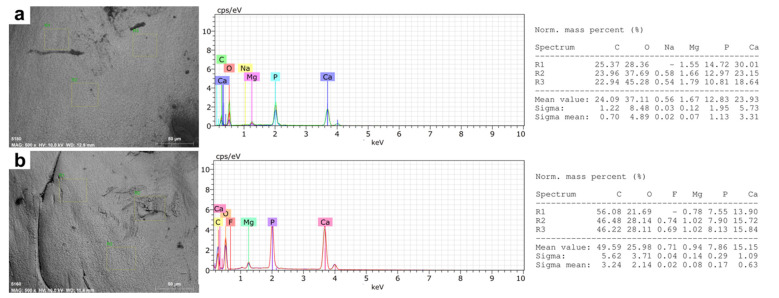
Elemental Microanalysis in teeth of rainbow trout *(Oncorhynchus mykiss)*. (**a**) Farmed fish. (**b**) Wild fish. BSD image, 500× magnification. Scanning Electron Microscope (Hitachi SU3500, Tokyo, Japan) coupled to XFlash^®^ Detector 410 and Quantax Esprit 1.8.1 Software controller (Bruker, Germany).

In the elemental microanalysis of the tooth samples, we observed differences between healthy vs. wild fish, mainly in calcium, phosphorous, and carbon percentages (Figure 3a,b).

The percentages of Ca and P between the samples of healthy fish (Ca: 23.78%; P: 11.7%; *p* = 0.001) and wild fish (Ca: 17.63%; P: 8.32%; *p* = 0.001), and in the latter an increase in the percentage of C (*p* < 0.001), representing almost 46.8% of the total composition of the teeth in wild fish (Table 3).

**Figure 4:**
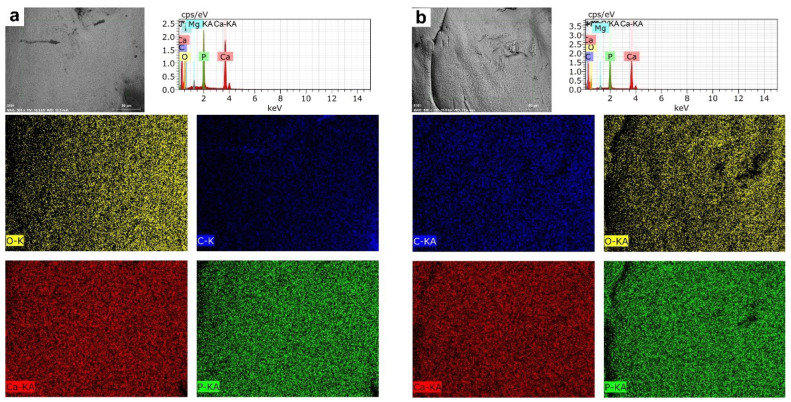
Mapping Elemental Distribution in teeth of rainbow trout *(Oncorhynchus mykiss).* (**a**) Farmed fish. (**b**) Wild fish. BSD image, 500× magnification. Scanning Electron Microscope (Hitachi SU3500, Tokyo, Japan) coupled to XFlash^®^ Detector 410 and Quantax Esprit 1.8.1 Software controller (Bruker, Germany).

The EDS mapping on farmed fish teeth showed a homogenous elemental distribution pattern. On the other hand, we observed the biggest microfractures in wild fish teeth showing a non-homogeneous pattern of elemental distribution for P, C, and O but not Ca (Figure 4a,b).

**Figure 5:**
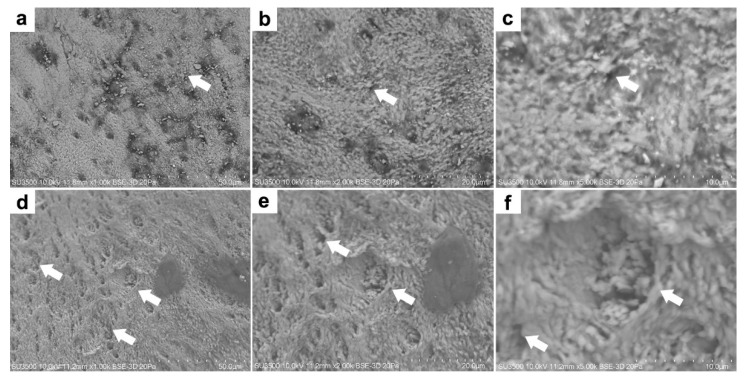
Microstructure of rainbow trout (*Oncorhynchus mykiss*) jawbone. Farmed fish at 1000×, 2000× and 5000× magnification (**a**–**c**). Wild fish at 1000×, 2000× and 5000× magnification (**d**–**f**). Backscatter detector (BSD) image. Scanning Electron Microscope SU3500, Hitachi, Tokyo, Japan.

In mandibular bone, the micrographs reveal the morphology of greater surface uniformity, rough and relatively defined, characteristic of HPA crystals. In addition, small ducts characteristic of the bone structure are observed (Figure 5a,d), which remains more accentuated when the region of interest is magnified (Figure 5b,c,e,f).

**Figure 6:**
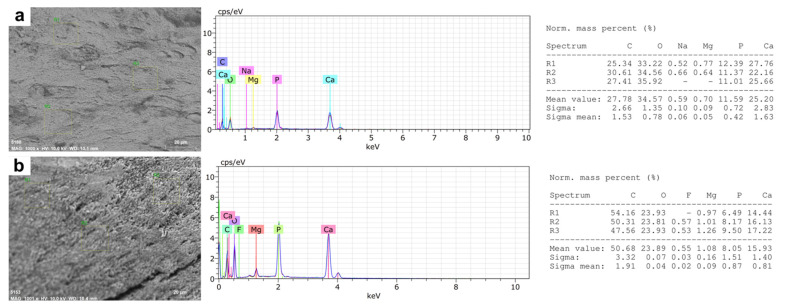
Elemental Microanalysis in the jawbone of rainbow trout *(Oncorhynchus mykiss)*. (**a**) Farmed fish. (**b**) Wild fish. BSD image, 1000× magnification. Scanning Electron Microscope (Hitachi SU3500, Tokyo, Japan) coupled to XFlash^®^ Detector 410 and Quantax Esprit 1.8.1 Software controller (Bruker, Germany).

Regarding the mineralization analysis, we observed differences between farmed fish and wild fish percentages, mainly calcium, phosphorous, and carbon concentration (Figure 6a,b).

The Ca and P percentages in farmed fish (21.9% and 10.1%, respectively), values that were higher than those observed in wild fish (14.1%; *p* = 0.009 and 6.83%; *p* = 0.012) (Table 4).

**Figure 7:**
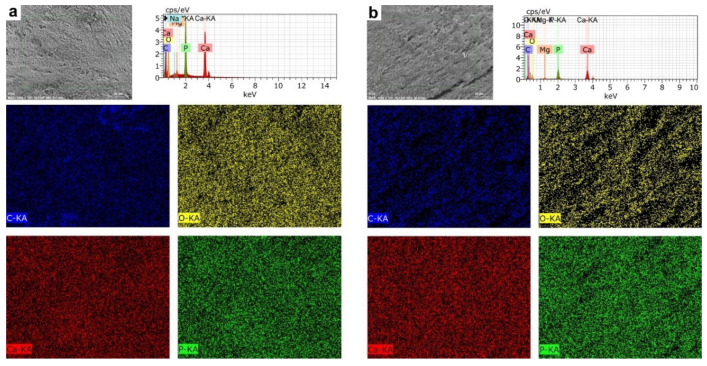
Mapping Elemental Distribution in the jaw bone of rainbow trout *(Oncorhynchus mykiss)*. (**a**) Farmed fish. (**b**) Wild fish. BSD image, 1000× magnification. Scanning Electron Microscope (Hitachi SU3500, Tokyo, Japan) coupled to XFlash^®^ Detector 410 and Quantax Esprit 1.8.1 Software controller (Bruker, Germany).

The EDS mapping in the mandibular bone showed microfractures, roughness, and pores in farmed and wild fish. These structure alterations were larger and more numerous in wild fish. We observed a more homogeneous elemental distribution pattern in farmed fish than in wild fish. The structure alterations affected P, C, and O but not Ca (Figure 7a,b).

**Figure 8:**
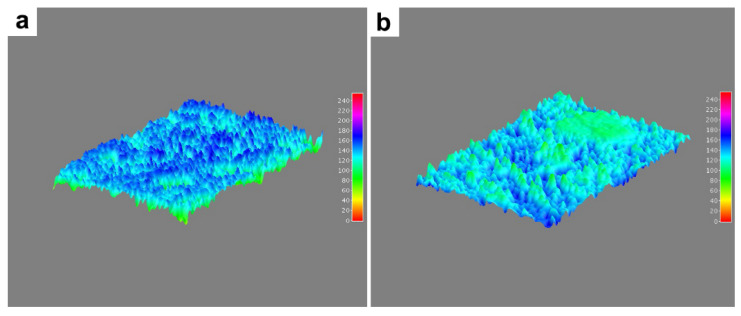
3D projection in rainbow trout (*Oncorhynchus mykiss*) jawbone microstructure. (**a**) Farmed fish. (**b**) Wild fish. Chemical contrast detector (BSD) image, 1000× magnification. ImageJ software 1.53a (National Institutes of Health, Bethesda, MA, USA, public domain).

We performed a 3D projection of the mandibular bone surface microstructure and porosity analysis (Figure 8). Findings show predominant areas of homogeneous mineralization in the mandibular bone of farmed fish (Figure 8a, blue). In contrast, wild fish (Figure 8b) showed a predominance of areas with hypomineralization (green).

Porosity analysis in the images showed more pores of larger sizes that covered a higher percentage of the region of interest in wild fish than in farmed fish (Table 5).

### 3.3. Thermogravimetric Analysis in Mandibular Bone (TGA-DSC)

**Figure 9:**
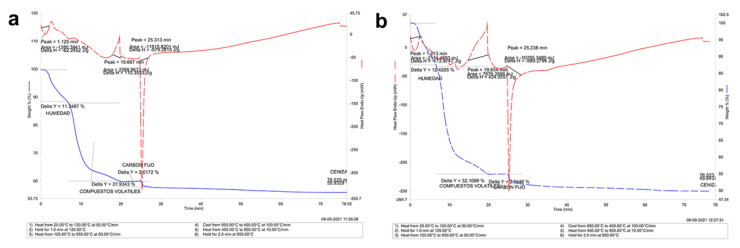
Thermogravimetric Analysis-TGA (Compositional) in mandibular bone. (**a**) Farmed fish. (**b**) Wild fish. TGA-DSC STA6000, Perkin Elmer, Waltham, MA, USA.

The proximal thermogravimetric analysis (Figure 9) showed the loss of mass (blue) due to the action of temperature (red) in the presence of oxygen or oxidative decomposition of the material. In both groups, we recorded three mass changes during the combustion time, where the times associated with mass changes were 1.12 min; 19.7 min, and 25.3 min (Figure 5a) in farmed fish; whereas in wild fish, it was 1.21 min; 19.7 min and 25.3 min (Figure 5b). The graph gives us moisture values of 11.3% and 12.4%; fixed carbon of 2.01% and 2.94%; carbon-derived volatile compounds of 27.93% and 42.83% and ash (minerals) of 55.9%, 49.8% in the mandibular bone of healthy and wild fish, respectively.

**Figure 10:**
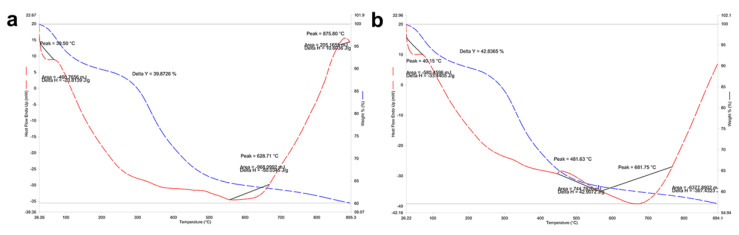
Differential Scanning Calorimetry DSC (transitions-purity) in mandibular bone. (**a**) Farmed fish. (**b**) Wild fish. TGA-DSC STA6000, Perkin Elmer, Waltham, MA, USA.

The differential scanning calorimetry or DSC analysis (Figure 10) describes changes in the sample associated with increased temperature in the absence of oxygen. The graphs show two curves: the mass loss curve (red curve) and water (blue curve) from the structure. As for the total mass loss was 39.87% and 42.83% for farmed and wild fish, respectively. This mass reduction or transitions occurred at three temperatures: 39.5 °C; 334.4 °C, and 628.7 °C for farmed fish (Figure 10a) and at 40.1 °C; 481.6 °C and 681.7 °C for wild fish (Figure 10b), with the most significant temperature difference in the second transition (>110 °C between one sample and the other).

## 4. Discussion

Metabolic analysis in blood samples is essential to evaluate the physiological status at different stages of development. In addition, it is a minimally invasive procedure that allows assessing the effect of nutritional status and the incorporation of microelements, vitamins, or proteins in the diet, measuring toxicity, immune status, or stress related to fish culture management [41,48]. Total protein content, enzymatic biomarkers of the functional status of different systems, and other serum markers such as cholesterol, triglycerides, and glucose are key to defining the nutritional status [41,49,50]. According to the data obtained, a low nutrient intake does not necessarily produce changes at the systemic level in fish, and they can even go without food for some time. The data obtained for systemic metabolites such as total proteins and albumin are concordant with what is reported in the literature for freshwater-farmed trout specimens [41,45,51]. In contrast, enzymatic biomarkers such as phosphatase and creatin-kinase vary from what is reported [41]. In comparison to our results, alkaline phosphatase and creatine kinase activities in juvenile trout were 608–1092 U/L and 3173–9800, respectively [41]. While, for presmolt-smolt (juveniles) alkaline phosphatase and creatine kinase values were <264 U/L and <10,177 U/L, respectively [45]. As for acid phosphatase, the values reported were 5.88 U/L in farmed and 13.3 U/L in wild fish; there are no previous reports for this protein in rainbow trout.

Glucose level is an essential nutritional parameter. Although it varies with age, its importance lies as a marker of stress in animals due to its relation to corticoid production [45,52]. The values obtained for healthy and wild fish agree with that reported for pre-smolt to smolt (juvenile) freshwater trout (0.2–4.1 mmol/L) [45]. Lower levels may occur in stages of reproductive activity, while higher levels can be related to stress [41,49,50]. We observed differences in glucose levels between farmed and wild fish. This finding could be related to higher stress found in farmed specimens, elevating serum glucose levels in response to increased cortisol and catecholamines, and elevating hepatic and muscle glycogenolysis activity [45,53]. However, considering that fish farming is regulated nationwide [54] and internationally [55] in specific matters such as the number of specimens per tank, food, and culture condition, further studies should address this difference to confirm its possible relation to the stress levels found in the fish.

Bone growth and metabolism is regulated by the quantity of microelements taken up from the water (Ca, P, Fe, Mg, Zn, and F). The low availability of these microelements translates into bone mineralization alterations [34,56,57,58]. Calcium and phosphorous play a vital role in the formation and remodeling of the inorganic bone matrix, as they are involved in normal skeletal growth and development by tissue mineralization [25,34,59]. At the bone level, calcium-binding is performed by osteocalcin, undergoing carboxylation at its glutamic acid residues through a vitamin K-dependent process, leading to both increased affinity of HPA for Ca and increased retention in the bone matrix [35,60,61]. Phosphorous uptake is only through diet and is regulated by phosphatases; alkaline phosphatases regulate the mineralization process through the fixation by precipitation of Ca-bound phosphate and further regulation of its release from phosphate esters [29]; whereas acid phosphatase produces dephosphorylation of matrix proteins, leading to phosphate release from bone tissue [29].

Different studies show that systemic markers of bone mineralization are Ca and P [34] and, indirectly, Fe and Mg [33,34,35,39]. We did not find microelements, such as Fe and Zn, in the mandibular bone or teeth. Serum and bone Mg were within average values, as the presence of Fe in bone. Differences observed were mainly in C, Ca, and P. Previous studies have reported that the Ca/P ratio serves as a mineralization parameter at the bone level [8,9,31,32].

The microstructural analysis of the mandibular bone—including teeth—from farmed and wild fish involves the visualization of morphology, porosity, and roughness. Our data showed more microfractures, pores, and defects in wild fish (Figure 2 and Figure 5), which may be a product of their free-living condition since there are no differences in nutritional parameters. Also, data obtained from micro elemental quantification using SEM-EDX revealed, in wild fish, greater defects in the microstructure, lower Ca/P mineral content and higher carbon percentage (Figure 3 and Figure 6), which is reflected in different elemental distribution (Figure 4 and Figure 7) and accentuated in the teeth above the mandibular bone (Table 3 and Table 4).

The TGA-DSC analysis showed similar transitions indicating purity, referring to the same type of biomaterial, i.e., there is no significant difference in the type of crystal that forms the bone. The compositional results correlated directly with the SEM-EDX analysis since the microstructure having more surface alterations in wild fish showed lower ash percentage (inorganic material) and higher content of fixed carbon and volatiles in farmed fish (Figure 9). It should be noted that there is a discrete difference regarding moisture percentage in the proximal TGA analysis (Figure 10), which is related to increased porosity in wild fish (Table 5). While farmed and wild fish showed a similar pore size, the number and total area in wild fish were higher than in farmed fish (Table 5). These results are consistent with 3D projection (Figure 8). In addition, the porosity percentage in wild fish does not represent an area greater than 6% of the coverage area analyzed. Although the porosity influences the strength and biomechanical properties of the bone, we do not know if it is significant due to a lack of reports on rainbow trout [62]. Considering these results and that both samples for TGA-DSC analysis were sprayed and oven-dried prior to analysis to remove surface water, it is not possible to attribute the difference in water retained within the sample to the porosity of the sample. We observed a relationship between Ca and P bioavailability and bone mineralization. However, this relationship is not entirely clear since information about bone homeostasis mechanisms is lacking. Our results indicate that the Ca/P ratio is maintained due to the stoichiometry of the HPA crystal, confirming that the Ca/P ratio changes at the systemic level, that Ca remains practically unchanged, and that the analyte most affected is systemic P since its increase in wild fish is due to an increase in Alkaline Phosphatase activity. Regarding the microstructure morphology, it is vital to highlight the porosity and roughness of HPA as a biomaterial since it favors cell adhesion [63,64].

## 5. Conclusions

To understand the mechanisms involved in bone tissue formation and remodeling processes, sustained availability of nutrients and microelements—such as calcium and phosphorus—is required. However, our results do not clarify the role of Fe and Mg bioavailability on bone microstructure. The Ca/P ratio in serum is not the same as in bone because phosphorus is structural in bone and a structural part of nucleic acids, proteins, lipids, and other molecules, such as high-energy phosphates. Therefore, it is required to ensure enough quantity for all these functions. Incorporating destructive and non-destructive techniques for the analysis of the microstructure of bone tissue is highly useful for understanding how slight structural and mineralization differences impact the mechanical/physicochemical properties for the correct functioning of the bone.

## Figures and Tables

**Table 1:** Blood metabolites in rainbow trout (*Oncorhynchus mykiss*) from fresh water.

	Medium (Minimum–Maximum)		*p*-Value
Blood Metabolites	Farmed Fish	Wild Fish	
Albumin (g/L)	30.0 (24.7–41.8)	18.1 (15.5–20.4)	<0.001
Acid Phosphatase (U/L)	5.88 (3.73–7.56)	13.3 (8.51–21.6)	<0.001
Alkaline Phosphatase (U/L)	229 (131–394)	594 (340–860)	<0.001
Creatine kinase (U/L)	7021 (513–15,920)	2192 (1045–3249)	0.024
Globulin (g/L)	37.6 (15.0–67.2)	28.9 (18.1–37.7)	0.053
Glucose (mmol/L)	4.49 (2.75–6.64)	3.43 (3.26–3.63)	<0.001
Total Protein (g/L)	67.6 (41.1–95.6)	47.0 (34.6–56.4)	<0.001

**Table 2:** Blood microelements in rainbow trout (*Oncorhynchus mykiss*) from fresh water.

	Medium (Minimum–Maximum)		*p*-Value
Blood Microelements	Farmed Fish	Wild Fish	
Calcium (mmol/L)	4.16 (2.70–5.90)	4.07 (3.60–4.50)	0.516
Phosphorus (mmol/L)	6.63 (4.60–8.60)	8.54 (6.80–11.40)	<0.001
Ca/P ratio	0.63 (0.49–0.81)	0.48 (0.39–0.60)	0.106
Iron (µmol/L)	15.19 (12.80–18.20)	13.28 (9.70–19.80)	0.001
Magnesium (mmol/L)	2.14 (0.89–4.32)	2.95 (2.68–3.28)	<0.001

**Table 3:** Semi-quantitative elemental microanalysis in teeth of rainbow trout (*Oncorhynchus mykiss*) by SEM-EDX.

	Medium (Minimum–Maximum)		*p*-Value
Mass Per Cent (%W)	Farmed Fish	Wild Fish	
Carbon	22.72 (18.31–35.31)	46.87 (37.38–56.08)	<0.001
Oxygen	39.44 (28.36–45.28)	25.70 (15.75–33.71)	<0.001
Calcium	23.78 (18.45–30.15)	17.63 (13.90–20.74)	0.001
Phosphorus	11.70 (7.90–14.72)	8.32 (5.89–10.57)	0.001
Ca/P ratio	2.06 (1.72–2.99)	2.17 (1.82–3.45)	0.637
Magnesium	1.40 (0.55–2.18)	0.89 (0.75–1.02)	0.094
Fluoride	0.59 (0.00–1.70)	0.81 (0.69–0.87)	0.73

**Table 4:** Semi-quantitative elemental microanalysis in mandibular bone of rainbow trout (*Oncorhynchus mykiss*) by SEM-EDX.

	Medium (Minimum–Maximum)		*p*-Value
Mass Per Cent (% W)	Farmed Fish	Wild Fish	
Carbon	31.31 (15.30–48.84)	44.61 (31.11–57.68)	0.009
Oxygen	34.47 (29.95–42.61)	29.91 (22.26–36.03)	0.049
Calcium	21.90 (12.60–29.26)	14.07 (9.20–17.98)	0.006
Phosphorus	10.11 (5.78–14.11)	6.83 (4.81–8.67)	0.012
Ca/P ratio	2.18 (1.81–2.50)	2.06 (1.73–2.32)	0.993
Magnesium	0.78 (0.00–1.11)	0.62 (0.52–0.78)	0.018
Fluoride	0.10 (0.00–0.92)	0.16 (0.00–0.64)	0.702

**Table 5:** Porosity analysis in jaw of rainbow trout (*Oncorhynchus mykiss*).

	Medium (Minimum–Maximum)		*p*-Value
Parameters	Farmed Fish	Wild Fish	
Pore Number	7940.83 (6281.92–9598.84)	11,339.60 (7071.97–15,607.23)	0.048
Total Area (µm^2^)	34.97 (22.69–47.25)	55.18 (32.19–78.17)	0.14
Pore Size (nm)	4.45 (3.42–5.48)	4.89 (3.19–6.57)	0.667
% of Total Area	3.46 (2.67–4.25)	5.81 (4.67–6.95)	0.261

## Data Availability

All data generated in this study have been included in this article.
